# Reductive Electropolymerization and Electrochromism of Iron(II) Complex with Styrene-Based Ligand

**DOI:** 10.3390/ma14174831

**Published:** 2021-08-25

**Authors:** Sergiusz Napierała, Kacper Muras, Grzegorz Dutkiewicz, Monika Wałęsa-Chorab

**Affiliations:** Faculty of Chemistry, Adam Mickiewicz Unversity in Poznań, Uniwersytetu Poznańskiego 8, 61-614 Poznań, Poland; sergiusz.napierala1999@gmail.com (S.N.); kacmur1@st.amu.edu.pl (K.M.); gdutkiew@amu.edu.pl (G.D.)

**Keywords:** reductive electropolymerization, iron(II) complex, N_3_-donor ligand, electrochromism

## Abstract

The benzimidazole-based ligand containing polymerizable styrene group has been prepared via condensation of picolinaldehyde derivative containing styrene moiety and benzimidazole-based hydrazine. The ligand reacted with iron(II) tetrafluoroborate and iron(II) trifluoromethanesulfonate giving red-brown complexes of Fe(II) ions of formula [Fe**L**_2_]X_2_, where X = CF_3_SO_3_^−^ (**1**) or BF_4_^−^ (**2**). Reductive electropolymerization was used to obtain a thin layer of the polymeric complex, **poly-1**. Further investigation of electrochemical properties of the compound by cyclic voltammetry showed two quasi-reversible redox processes assigned to electrooxidation and electroreduction of the polymer. Spectroelectrochemical measurements confirmed that the polymer undergoes the color changes during oxidation and reduction process. The polymer in its neutral state (Fe(II)) is yellow and it exhibits absorption band at 370 nm, after oxidation to Fe(III) state absorption band shifts to 350 nm and the polymer is almost colorless. While the metal ions are reduced to Fe(I) absorption band at around 410 nm has been observed and the polymer changed its color to intense yellow. The stability of the polymer during multiple oxidation/reduction cycles has also been investigated.

## 1. Introduction

Electropolymerization it is the well-known method of formation of thin, insoluble layers of polymeric materials, in which the formation of the polymer occurs directly on the surface of the conductive substrates [1]. The advantage of the use of electropolymerization as a method of formation of thin films is that only a good solubility of the monomers is required and this way the solubility problem of the polymer is avoided. By adjusting the duration of the electropolymerization the surface coverage and film thickness can be easily controlled. Electropolymerized films are adhesive, stable and electrochemically active. It is also possible to perform the electrochemical copolymerization of monomers, which is highly effective for producing multicolor electrochromic polymers [2,3,4]. Because of these features, electropolymerization has become an important method for making thin films for applications in various electronic devices.

Although oxidative electropolymerization occurs in the majority of cases [5,6,7,8], reductive electropolymerization that undergoes on the cathode is also possible. Reductive electropolymerization has been found to be an efficient method of formation of thin films of polymers derived from pyridyl and polypyridyl complexes containing styrene groups [9,10,11,12,13,14,15]. The mechanism of reductive electropolymerization of complexes containing vinyl-substituted ligands is believed to be anionically initiated [16,17], followed by C–C bond formation as a result of the reaction between formed radicals and side-by-side chain propagation [18]. The reductive electropolymerization of vinyl-substituted complexes has been also useful for the copolymerization of various metal complexes [11,19]. The polymerization of compounds containing styrene groups leading to formation of thin films on the electrode surface can also be done by radical polymerization reaction [20,21] but it usually requires the use of some additional initiators, such as AIBN or temperature to initiate the formation of free radicals. 

Hydrazone N_3_-donor ligands are known to be analogues of terpyridines having the same coordination preferences and forming complexes of similar structures. The advantage of hydrazone ligands over their terpyridine counterparts is a straightforward synthesis via condensation reaction of aldehydes or ketones with appropriate hydrazine derivative, while the terpyridines are usually obtaining via multistep condensation or coupling reactions [22,23,24]. Besides being easier to synthesize, hydrazone ligands, due to this fact that the only one byproduct of the condensation reaction is water, can be purified by simple crystallization in comparison to the terpyridines that are usually purifying by column chromatography. Also, the synthesis of both hydrazones and Schiff-base ligands usually occur in ethanol decreasing the use of toxic and harmful organic solvents [25]. 

Herein we present the synthesis of hydrazone N_3_-donor ligand and its complexes with Fe(II) ions. The complexes have been designed to possess two styrene groups that undergo reductive electropolymerization during multiple electrochemical cycles. Complexes have been characterized by spectroscopic methods, as well as by X-ray diffraction analysis confirming the 1:2 metal: ligand stoichiometry. The complex **1** has been successfully electropolymerized on the ITO electrode surface and its electrochemical and electrochromic properties have been investigated. 

## 2. Materials and Methods

### 2.1. General

The aldehyde **A** and the benzimidazole derivative **B** have been obtained according to a previously reported procedures [20,26,27]. NMR spectra were recorded on a Bruker UltraShield 300 MHz spectrometer (Bruker Corporation, Billerica, MA, USA) and were calibrated against the residual protonated solvent signal (d_6_-DMSO, δ 2.50 ppm). High-resolution mass spectra were run on a QTOF spectrometer (Impact HD Brucker, Bruker Daltonics, Bremen, Germany) in positive ion mode. A conventional three-electrode cell was used for electrochemical measurements with a platinum electrode as the working electrode, a platinum wire as the counter electrode and a non-aqueous Ag/Ag^+^ electrode as the reference electrode. The supporting electrolyte was 0.1 M solution of tetrabutylammonium perchlorate (TBAClO_4_) in acetonitrile. The acetonitrile for the preparation of the supporting electrolyte was dried by passing over neutral alumina followed by the storage over 3 Å molecular sieves [28]. The electrolyte before measurements was purged with argon for 20 min. to remove the dissolved oxygen and the argon blanket was maintained over the solution to prevent oxygen diffusion during measurements. The cyclic voltammograms were obtained using a VSP multichannel potentionstat (BioLogic, Seyssinet-Pariset, France). The spectroelectrochemical measurements in solution were done using commercially available honeycomb electrode (Pine Research Instrumentation, Inc., Durham, NC, USA). The spectroelectrochemistry of the polymer was done using a polymer modified indium-tin oxide coated (ITO) glass slides, Pt wire as a counter electrode and a silver wire as a pseudoreference electrode. The SEM images were done on the scanning electron microscope (Quanta 250 FEG, Thermo Fisher Scientific wissenschaft-liche Geräte GmbH, Wien, Austria), AFM images were done using atomic force microscope (Agilent 5500, Agilent Technologies Ltd., Yarnton, UK).

### 2.2. Synthesis

Ligand **L**: To a solution of aldehyde **A** (0.25 g, 1.2 mmoL) in ethanol (10 mL), benzimidazole derivative **B** (0.19 g, 1.2 mmoL) was added. The mixture was stirred at room temperature under an argon atmosphere overnight. The crude product was isolated by evaporation of the solvent. Afterward, the residue was solubilized in a minimum volume of dichloromethane and the final product was obtained via crystallization by gradual addition of hexane. Yield: 62.31% (0.2638 g). ^1^H NMR (300 MHz, DMSO) δ 11.91 (s, 1H), 8.40 (dd, *J* = 7.2, 1.6 Hz, 1H), 8.15 (d, *J* = 8.3 Hz, 2H), 7.99–7.85 (m, 3H), 7.61 (d, *J* = 8.2 Hz, 2H), 7.45–7.31 (m, 2H), 7.10–7.00 (m, 2H), 6.81 (dd, *J* = 17.6, 11.0 Hz, 1H), 5.93 (dd, *J* = 17.7, 1.0 Hz, 1H), 5.33 (dd, *J* = 10.8, 1.0 Hz, 1H), 3.73 (s, 3H) ppm. ^13^C NMR (75 MHz, DMSO) δ 155.1, 154.1, 153.4, 142.7, 137.9, 137.7, 137.5, 137.0, 136.2, 134.2, 126.8, 126.5, 120.8, 120.1, 119.4, 118.5, 116.5, 115.1, 31.8 ppm. HR-MS (ESI) *m*/*z*: [L+H]^+^ calcd. for C_22_H_20_N_5_ 354.1714; found 354.1716; [L+Na]^+^ calcd. for C_22_H_19_N_5_Na 376.1533; found 376.1537. Elemental analysis calcd. for C_22_H_19_N_5_ C, 74.77; H, 5.42; N, 19.82; found C, 74.75; H, 5.48; N, 19.84. 

Synthesis of complexes-general procedure: A mixture of ligand **L** and an appropriate iron(II) salt in 1:2 ligand to metal molar ratio was solubilized in a dichloromethane/acetonitrile mixture 2:1 (*v*/*v*) and the solution was stirred at room temperature for 24 h under the normal atmosphere. The solution was concentrated and diethyl ether was added to precipitate the complex. The obtained solid was centrifuged, washed with diethyl ether and dried.

Complex **1**: Obtained as a dark-red solid. Yiled: 69% (42 mg). HR-MS (ESI) *m*/*z*: [Fe**L**_2_]^2+^ calcd. for C_44_H_38_N_10_Fe 381.1310; found 381.1306; [Fe**L**(**L**−H)]+ calcd. for C_44_H_37_N_10_Fe 761.2547; found 761.2546. Elemental analysis calcd. for Fe(C_22_H_19_N_5_)(CF_3_SO_3_)_2_ C, 52.08; H, 3.61; N, 13.20; S, 6.05; found C, 52.14; H, 3.63; N, 13.19; S, 6.04.

Complex **2**: Obtained as a red solid. Yield: 63% (25 mg). HR-MS (ESI) *m*/*z*: [Fe**L**_2_]^2+^ calcd. for C_44_H_38_N_10_Fe 381.1310; found 381.1315. Elemental analysis calcd. for Fe(C_22_H_19_N_5_)(BF_4_)_2_ C, 56.44; H, 4.09; N, 14.96; found C, 56.43; H, 4.14; N, 14.93.

### 2.3. X-ray Crystallography

Diffraction data were collected by the ω-scan technique, using graphite-monochromated MoK_α_ radiation (λ = 0.71073 Å), at room temperature (**1**) and at 100(1) K (**2**) on Rigaku Xcalibur four-circle diffractometer with EOS CCD detector (Agilent Technologies Ltd., Yarnton, UK). The data were corrected for Lorentz-polarization as well as for absorption effects [29]. Precise unit-cell parameters were determined by a least-squares fit of the reflections of the highest intensity, chosen from the whole experiment. The structures were solved with SHELXT [30] and refined with the full-matrix least-squares procedure on F^2^ by SHELXL [31]. All non-hydrogen atoms were refined anisotropically. Hydrogen atoms were placed in idealized positions and refined as ‘riding model’ with isotropic displacement parameters set at 1.2 (1.5 for CH_3_) times U_eq_ of appropriate carrier atoms. The crystals of **1** has been twinned, and this was taken into account both during date reduction [29] and structure refinement [30]; the BASF factor, related to the ratio of both components, refined at 0.3553(17). In the structure of **2** large voids have been found, filled with diffused electron density—probably highly disordered solvent. As the attempts to model the reasonable solvent model failed, the SQUEEZE procedure has been successfully applied. Crystal data, data collection and structure refinement are shown in Table 1.

## 3. Results and Discussion

**Figure 1:**
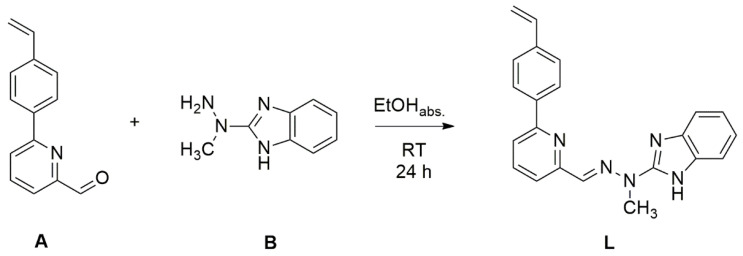
Synthetic scheme of preparation of **L**.

The ligand **L** has been obtained via condensation reaction between picolinaldehyde containing styrene group **A** and benzimidazole-based hydrazine **B** as outlined in Figure 1.

The reaction was carried out in an absolute ethanol for 24 h at room temperature. The ligand **L** has been obtained as a slightly yellow crystalline solid with the yield of 63% via crystallization from a dichloromethane/hexane. The ligand **L** has been used in complexation reactions with two different salts of Fe(II) ions: tetrafluoroborate and trifluoromethanesulfonate. The complexation reactions were done at room temperature for 24 h in dichloromethane/acetonitrile 2:1 (*v*/*v*) mixture and the complexes of stoichiometry metal:ligand 1:2 have been obtained as red-brown colored solids via evaporation of the solvent and precipitation from a minimum volume of acetonitrile by gradual addition of diethyl ether. The complexes have been characterized using spectroscopic methods. In the ESI-MS spectra of complexes peaks at *m*/*z* = 381 and 761 have been observed that are associated with the presence of [Fe**L**_2_]^2+^ and [Fe**L**(**L**−H)]^+^ molecular cations in solution. The ESI spectra of complexes contain also the signals at *m*/*z* = 354 and 376 that have been assigned to molecular cations [**L**+H]^+^ and [**L**+Na]^+^. The presence of these signals suggest the instability of investigated complexes under ESI conditions.

**Figure 2:**
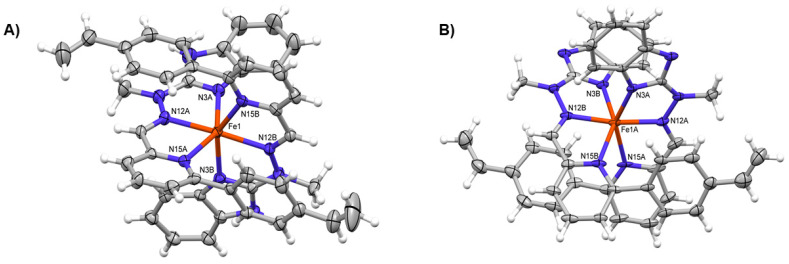
Perspective views of the cation observed in the structure **1** (**A**) and one of the symmetry-independent cationic complexes of **2** (**B**). Ellipsoids are drawn at the 50% probability level, hydrogen atoms are shown as spheres of arbitrary radii.

The unambiguous structures of complexes have been investigated by X-ray crystallography. The single crystals appropriate for X-ray measurements were grown by slow diffusion of diethyl ether into acetonitrile solutions of complexes. Figure 2 shows the perspective views of the dications **1** and **2**; Table 2 lists the relevant geometrical parameters. As expected, all complexes are of M**L**_2_ structure, and the overall shapes and coordinations of all three dications (in the structure **2** there are two symmetry-independent complexes) are very similar (Figure 2). The coordination of metal cation is best described as (highly) distorted octahedral one (cf. Table 2), with quite linear N12-Fe-N12 angle.

**Figure 3:**
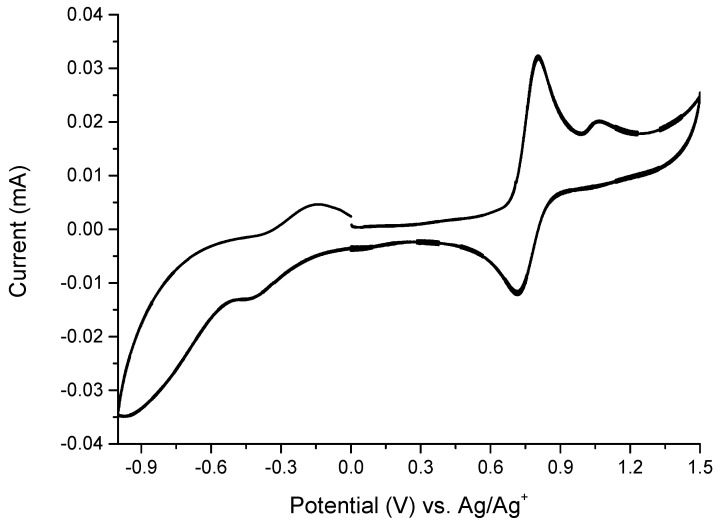
Cyclic voltammogram of complex **1** measured in anhydrous and deaerated acetonitrile with 0.1 M TBAClO_4_ as a supporting electrolyte at scan rate 100 mV·s^−1^.

To examine the usefulness of complexes as active materials in electronic devices they have been investigated in terms of their electrochemical properties. The solution electrochemical data for complexes **1** and **2** were obtained by cyclic voltammetry. The measurements were done in the three-electrode configuration in deaerated, anhydrous acetonitrile solution of tetrabutylammonium perchloride (0.1 M) as a supporting electrolyte. In case of complex **1** the two quasi-reversible oxidation/reduction waves have been observed with the half-wave potentials of −0.29 V and +0.76 V (Figure 3).

The two electrochemical processes can be assigned to redox couples [Fe(I)**L**_2_]^+^/[Fe(II)**L**_2_]^2+^ and [Fe(II)**L**_2_]^2+^/[Fe(III)**L**_2_]^3+^, respectively [32,33,34]. One additional irreversible wave assigned to the oxidation of the ligand molecule has been observed at +1.07 V in the anodic scan. In case of complex **2** the quasi-reversible redox wave at E_1/2_ = +0.76 V assigned to the oxidation of the metallic center has been observed. In the cathodic part of the cyclic voltammogram, the small quasi-reversible wave at E_pc_ = −0.18 V was noticed. This wave can be attributed to Fe(II)/Fe(I) redox couple (Figure S1). It has been known that when the value of i_pc_/i_pa_ is closer to 1 at the same scan rate, a more reversible reaction is expected [35]. As calculated for Fe(II)/Fe(III) redox couple the i_pc_/i_pa_ value for complexes **1** and **2** were found to be 0.7 and 0.3, respectively. The i_pc_/i_pa_ values lower than 1 indicate that the oxidation product is unstable and decompose. Due to complex **1** exhibited better electrochemical reversibility than **2**, it has been chosen for further investigations of electrochemical and electrochromic properties. To improve the reversibility of Fe(II)/Fe(III) redox couple we investigated the electrochemical properties on complex **1** in the narrower potential window up to +1.0 V to avoid the oxidation of ligand molecules (Figure S2). As expected, the reversibility of the anodic process increased and the i_pc_/i_pa_ value was calculated to be 0.85, what indicates that the decomposition of the complex during redox reaction was partially caused by the decomposition of oxidized ligand molecule. 

**Figure 4:**
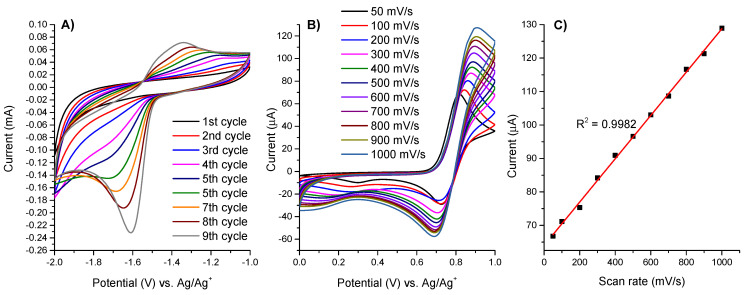
(**A**) Reductive electropolymerization of **1** in 0.1 M solution of TBAClO_4_ in deaerated and anhydrous acetonitrile on ITO glass electrode by 10 repeated cyclic potential scans between −1.0 V and −2.0 V at scan rate of 100 mV/s; (**B**) CV profiles of the **poly-1** film obtained at different scan rates; (**C**) Linear dependence on the scan rates of the anodic peak currents of the **poly-1** film.

When the potential was scanned repeatedly between −1.0 V and −2.0 V, the current increased gradually and continuously (Figure 4A). This indicates the in situ deposition of polymeric film on the electrode surface as a result of reductive electropolymerization of vinyl groups. It was observed that the cathodic peak currents of the forward scans are much higher that the anodic peak currents of the reverse scans. This is probably due to the partial decomposition of the reduced monomers but, despite this, the formation of thin layer of polymers on the electrode surface has been observed. The electrode was rinsed with copious amounts of CH_3_CN and then measured in a clean supporting electrolyte solution (Figure S3). The well-defined Fe(II)/Fe(III) and Fe(II)/Fe(I) couples are retained and the potential remains basically unchanged after polymerization similarly as observed for vinyl-containing polypyridyl ruthenium complexes [11]. To investigate whether the redox process is controlled by diffusion or adsorption the cyclic voltammograms of **poly-1** at different scan speeds were recorded (Figure 4B). The shift of the anodic peak potentials towards more negative potentials upon increasing the scan rate was observed, and similarly the cathodic peak shifted towards more positive potential with the increase of the scan speed. This indicates the presence of the electrochemical irreversibility and the quasi-reversible redox process. As revealed in Figure 4C, when the scan rate was different from 50 to 1000 mV/s, a linear dependence with the linear regression coefficient of R^2^ = 0.9982 of the redox response upon the scan rate (*v*) was detected, and it indicates that the redox events occur at the electrode surface [36].

**Figure 5:**
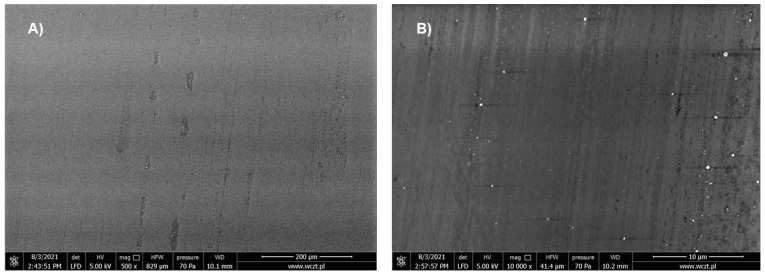
SEM image of the thin film of polymer **poly-1** with magnification 500× (**A**) and 10,000× (**B**).

The electropolymerization of **1** on ITO electrode produced the formation of adherent polymeric film. The morphology of the formed layer was investigated by scanning electron microscopy (SEM) and atomic force microscopy (AFM). As seen in Figure 5 the surface of the polymeric thin film is smooth and flat except for the presence of small irregular domains. 

**Figure 6:**
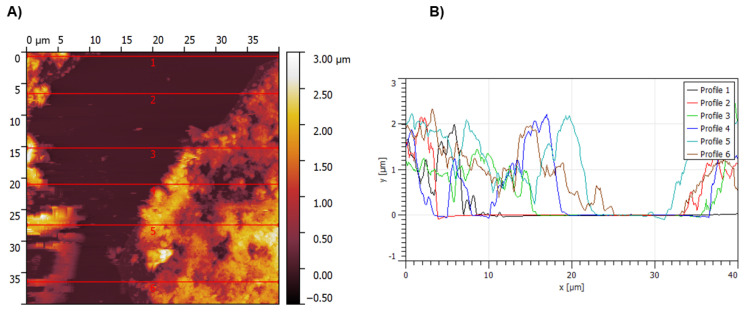
(**A**) AFM micrographs of **poly-1** at a 40 µm × 40 µm resolution scale deposited on ITO electrodes showing the step between the ITO and the polymer surface. (**B**) AFM cross-section profiles measured at a marked places.

The film thickness was investigated using AFM scratch method. To do this, the polymeric film was cutted with the blade and the difference between the polymeric film and bare ITO surface was measured in few different places by AFM (Figure 6). 

The film was found to have the average thickness of ~1.5 μm. Root mean square (RMS) roughness parameter measured by atomic force microscopy (AFM) was calculated to be 153 nm. 

**Figure 7:**
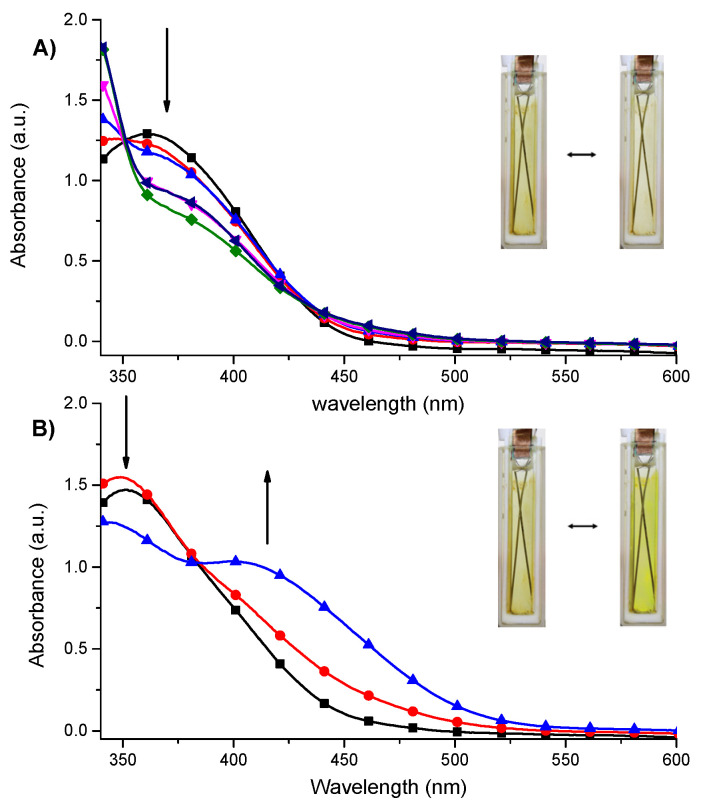
Spectroelectrochemistry of **poly-1** immobilized on ITO electrode measured in anhydrous and deaerated 0.1 M solution of TBAClO_4_ in acetonitrile as a supporting electrolyte with applied potentials of (**A**) 0 (■), +500 (●), +600 (▲), +700 (▼), +800 (◄) and +900 (♦) mV held for 30 s per potential; insert: the photograph of **poly-1** in its neutral (left) and electrochemically oxidized (right) states; (**B**) −100 (■), −200 (●) and −300 (▲) mV held for 30 s per potential; insert: the photograph of **poly-1** in its neutral (left) and electrochemically reduced (right) states.

The polymer **poly-1** was further investigated by spectroelectrochemistry in terms of the color change in response to an electric stimulus and its electrochromic properties has been compared with the properties of monomer **1** in solution. The advantage of spectrolectrochemistry is the cross-correlation of the information from both electrochemical and optical measurements and it allows to investigate the absorption changes resulting from species produced or consumed in the electrochemical process [37]. For both **1** in solution and **poly-1** the visible color changes have been observed during oxidation and reduction process (Figure 7 and Figure S4).

The complex **1** in Fe(II) state exhibits one absorption band at 370 nm attributed to metal-to-ligand charge transfer (MLCT). Upon electropolymerization, a slight blue shift (~7 nm to 363 nm) of this band was observed for the surface-grafted **poly-1** in comparison to monomer **1** in solution. It is probably due to conversion of the electron-withdrawing vinyl groups in **1** to saturated alkyl groups in the polymeric network during the reductive electropolymerization process [14]. When the positive potential was applied to **poly-1** the disappearance of the MLCT band was observed (Figure 7A). It was concomitant with the formation of ligand-to-metal charge transfer (LMCT) band with the absorption maxima in the ultraviolet region, below 350 nm [38,39,40]. It was the result of electrooxidation of Fe(II) ions and formation of the Fe(III) complex on ITO surface. The sharp isosbestic point at 350 nm indicates the presence of only neutral and oxidized species in the polymeric film and their independence. During this process the color of the film changed from yellow to almost colorless. After applying of negative potential to **poly-1** the formation of the band at around 410 nm was observed as a result of the reduction of Fe(II) to Fe(I) ions (Figure 7B). The **poly-1** in its reduced form was found to exhibit intense yellow color. Similar optical changes have been observed for **1** in solution (Figure S4). The absorption spectra of **poly-1** in its different redox states have been shown in Figure S5.

**Figure 8:**
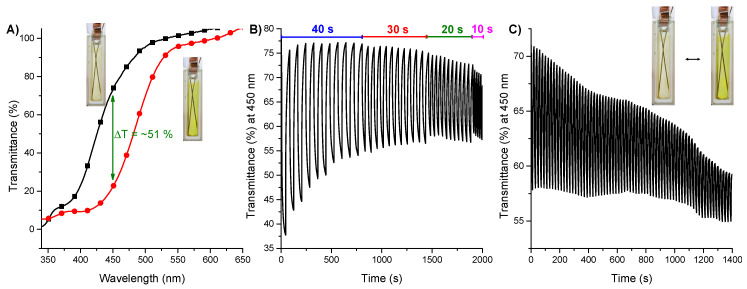
(**A**) The transmittance spectra of the **poly-1** in oxidized (black) and reduced (red) form; (**B**) Electrochromic switching between oxidized and reduced form of **poly-1** with intervals of 40, 30, 20 and 10 s monitored at 450 nm; (**C**) The Transmittance difference of **poly-1** by switching between oxidized and reduced states with 10 s intervals.

To investigate the electrochromic properties of **poly-1** in details, a chronoamperometry coupled with the UV-Vis spectroscopy was used to evaluate the long term stability of the film, switching time, optical contrast and coloration efficiency. The biggest difference in transmittance of oxidized and reduced states of **poly-1** was found to be 51% at the wavelength of 450 nm (Figure 8A). The changes in ΔT% values were measured by switching the potential between +1.0 V and −0.5 V with the time intervals from 40, 30, 20 to 10 s to assay the influence of the retention times on the contrast ratios of the polymer (Figure 8B). For **poly-1** the contrast ratio at 450 nm decreased from 50% to 23.1% after 10 switching cycles with 40 s interval, what indicates the gradual degradation of the polymeric film during multiple electrochemical processes. Then, the transmittance difference decreased to 22% when the intervals was changed to 30 s, through 16.5% for 20 s intervals to 13.3% for 10 s intervals. The **poly-1** was found to be stable during switching with 20 s and 10 s intervals; the transmittance difference did not change after 10 oxidation/reduction cycles. To further investigate the long-term stability of the **poly-1** potential was switched between +1.0 V and −0.5 V with 10 s intervals and the transmittance at 450 nm was investigated. As seen in Figure 8C the transmittance difference dropped down from 13.3% to 5% after 70 oxidation/reduction cycles (1400 s), what was probably caused the degradation of the polymer. The improvement of the stability of the layer for electronic applications could be achieved by increasing the degree of the cross-linking [41,42,43].

## 4. Conclusions

The terpyridine-like N_3_-donor ligand have been obtained via condensation of styrene-substituted picolinaldehyde and benzimidazole-based hydrazine. The self-assembly of the ligand with Fe(II) ions led to formation of mononuclear complexes of metal:ligand ratio 1:2. The complex [Fe**L**_2_](CF_3_SO_3_)_2_ was found to undergo reductive electropolymerization on the electrode surface forming the thin layer of polymer **poly-1**. Both the monomer and the polymer were found to undergo two quasi-reversible electrochemical processes with the visible color change from yellow to almost colorless during electrooxidation and to intense yellow during electroreduction of the metallic center. The color contrast of the polymer was investigated by switching between oxidized and reduced form (from almost colorless to bright yellow) with different time intervals from 40 s to 10 s. The long-term stability of the film was investigated and it was found that the polymer partially decompose during multiple oxidation/reduction cycles.

## Figures and Tables

**Table 1:** Crystal data, data collection and structure refinement.

Compound	1	2
Formula	C_44_H_38_FeN_10_^2+^ 2CF_3_SO_3_^−^·C_2_H_6_O	2(C_44_H_38_FeN_10_)^2+^ 4BF_4_^−^·5C_2_H_3_N
Formula weight	1106.90	2073.86
Crystal system	hexagonal	monoclinic
Space group	P6_3_	Cc
a (Å)	21.3035 (3)	31.2280 (10)
b (Å)	21.3035 (3)	11.6807 (5)
c (Å)	21.4739 (3)	29.1181 (17)
α (°)	90	90
β (°)	90	92.196 (4)
γ (°)	120	90
V (Å^3^)	8440.0 (3)	10,613.5 (8)
Z	6	1
D_x_ (g cm^−3^)	1.307	1.298
F (000)	3420	4264
μ (mm^−1^)	0.417	0.358
Reflections:		
collected	72,901	22,167
unique (R_int_)	68,984 (0.0334)	14,487 (0.0458)
with I > 2σ(I)	60,314	8953
R(F) [I > 2σ(I)]	0.0534	0.0722
wR(F^2^) [I > 2σ(I)]	0.1353	0.1789
R(F) [all data]	0.0630	0.1141
wR(F^2^) [all data]	0.1420	0.2017
Goodness of fit	1.055	1.092
max/min Δρ (e·Å^−3^)	0.91/−0.25	0.45/−0.61
CCDC deposition number	2,081,073	2,081,074

**Table 2:** Relevant geometrical parameters (Å, °) with s.u.’s in parentheses.

	1	2A	2B
Fe-N3	2.142(6) 2.153(6)	2.137(7) 2.145(7)	2.131(7) 2.133(8)
Fe-N12	2.150(7) 2.161(7)	2.140(10) 2.159(10)	2.117(9) 2.126(10)
Fe-N15	2.202(7) 2.281(7)	2.257(8) 2.266(8)	2.244(7) 2.269(8)
Angles	166.6(2) 146.7(2) 145.9(2)	171.9(3) 146.4(4) 146.0(4)	171.1(3) 145.6(3) 145.9(4)

## Data Availability

Data is contained within the article.

## Supplementary Materials

Crystallographic data for the structural analysis have been deposited with the Cambridge Crystallographic Data Centre. Copies of this information may be obtained free of charge from: The Director, CCDC, 12 Union Road, Cambridge, CB2 1EZ, UK; e-mail: deposit@ccdc.cam.ac.uk, or www.ccdc.cam.ac.uk. The following are available online at https://www.mdpi.com/article/10.3390/ma14174831/s1, Figure S1: Cyclic voltammogram of complex **2** measured in anhydrous and deaerated acetonitrile with 0.1 M TBAClO_4_ as a supporting electrolyte at scan rate 100 mV·s^−1^, Figure S2. Cyclic voltammogram of complex **1** measured in anhydrous and deaerated acetonitrile with 0.1 M TBAClO_4_ as a supporting electrolyte at scan rate 100 mV·s^−1^ in the potential window from 0 to +1.0 V, Figure S3. Cyclic voltammogram of **poly-1** measured in anhydrous and deaerated acetonitrile with 0.1 M TBAClO_4_ as a supporting electrolyte at scan rate 100 mV·s^−1^, Figure S4. Spectroelectrochemistry of **1** measured in anhydrous and deaerated 0.1 M solution of TBAClO_4_ in acetonitrile as a supporting electrolyte with applied potentials of (A) 0 (■), +500 (●), +600 (▲), +700 (▼), +800 (♦) and +900 (◄) mV held for 30 s per potential; insert: the photograph of **1** in its neutral (left) and electrochemically oxidized (right) states; (B) 0 (■), −200 (●), −300 (▲), −400 (▼), −500 (♦) and −600 (◄) mV held for 30 s per potential; insert: the photograph of **1** in its neutral (left) and electrochemically reduced (right) states, Figure S5. The absorption spectra of **poly-1** in its neutral Fe(II) state (■), oxidized Fe(III) state at +1.0V (●) and reduced Fe(I) state (▲) at −0.5V, Figure S6. ^1^H NMR of ligand **L** in d_6_-DMSO, Figure S7. ^13^C NMR spectra of ligand **L** in d_6_-DMSO, Figure S8. HR-MS spectra of ligand **L**, Figure S9. ESI-MS spectra of complex **1**, Figure S10. ESI-MS spectra of complex **2**.
