# Supplementary material for: Reductive Electropolymerization and Electrochromism of Iron(II) Complex with Styrene-Based Ligand
Source: Materials (Basel). 2021 Aug 25;14(17):4831. doi: 10.3390/ma14174831 (PMC8432686; doi:10.3390/ma14174831)
Supplement: Supplementary file 1 [file materials-14-04831-s001.zip › materials-1350488-supplement-proofed-checked.pdf]

materials

MDPI

Supplementary Information

Reductive Electropolymerization and Electrochromism of Iron(II) Complex with Styrene-Based Ligand

Sergiusz Napierała, Kacper Muras, Grzegorz Dutkiewicz and Monika Wałęsa-Chorab *

Faculty of Chemistry, Adam Mickiewicz Unversity in Poznań, Uniwersytetu Poznańskiego 8, 61-614 Poznań, Poland; sergiusz.napierala1999@gmail.com (S.N.); kacmur1@st.amu.edu.pl (K.M.); gdutkiew@amu.edu.pl (G.D.)
* Correspondence: mchorab@amu.edu.pl

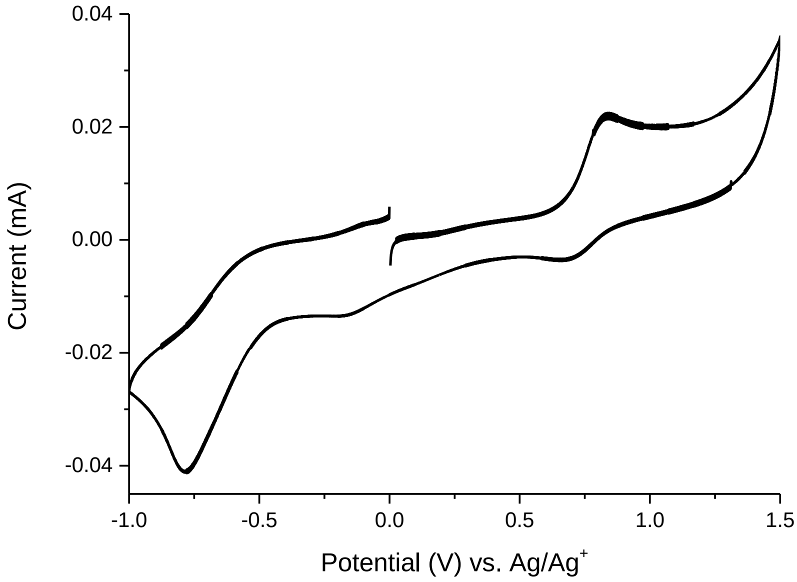

Figure S1. Cyclic voltammogram of complex **2** measured in anhydrous and deaerated acetonitrile with 0.1 M $TBAClO_4$ as a supporting electrolyte at scan rate 100 $mV \cdot s^{-1}$.

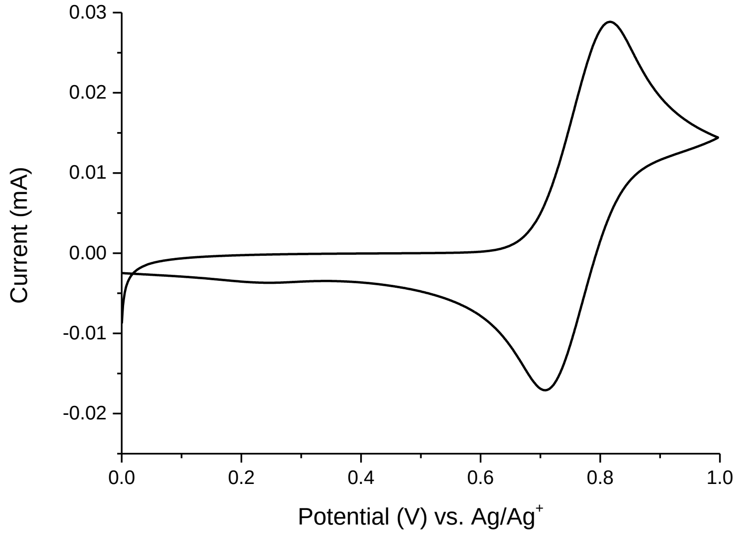

Figure S2. Cyclic voltammogram of complex **1** measured in anhydrous and deaerated acetonitrile with 0.1 M $TBAClO_4$ as a supporting electrolyte at scan rate 100 $mV \cdot s^{-1}$ in the potential window from 0 to +1.0 V.

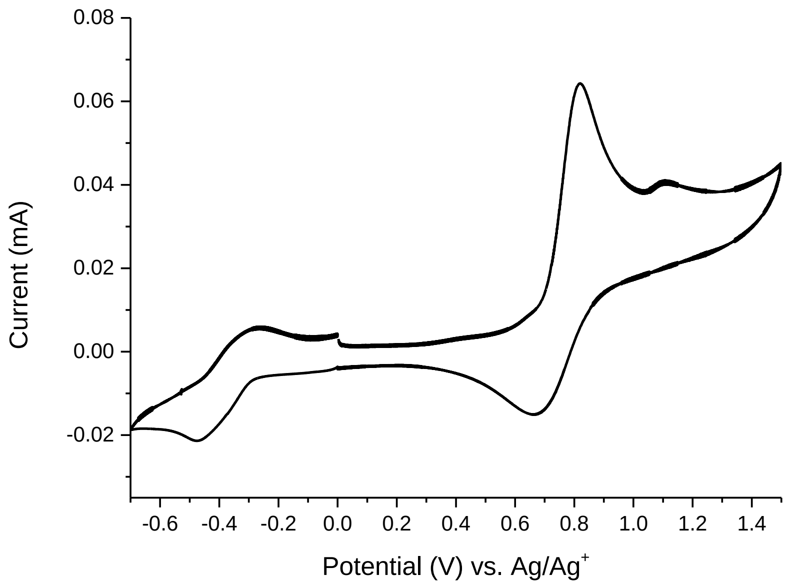

Figure S3. Cyclic voltammogram of **poly-1** measured in anhydrous and deaerated acetonitrile with 0.1 M $TBAClO_4$ as a supporting electrolyte at scan rate 100 mV·s^{-1}.

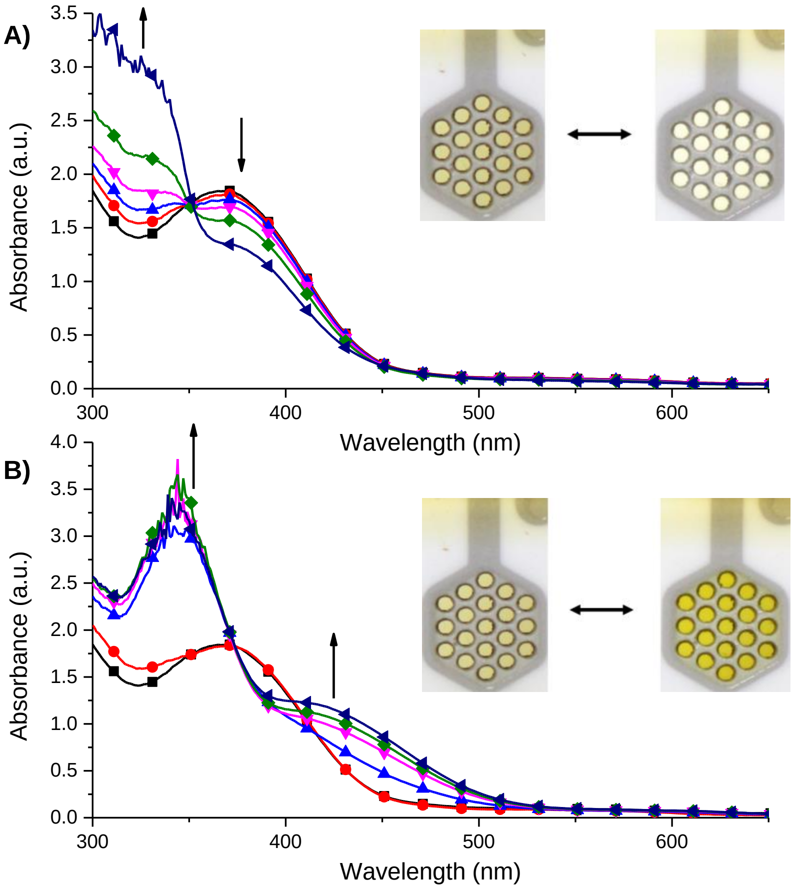

Figure S4. Spectroelectrochemistry of **1** measured in anhydrous and deaerated 0.1 M solution of $TBAClO_4$ in acetonitrile as a supporting electrolyte with applied potentials of A) 0 (■), +500 (●), +600 (▲), +700 (▼), +800 (◆) and +900 (◀) mV held for 30 s per potential; insert: the photograph of **1** in its neutral (left) and electrochemically oxidized (right) states; B) 0 (■), -200 (●), -300 (▲), -400 (▼), -500 (◆) and -600 (◀) mV held for 30 s per potential; insert: the photograph of **1** in its neutral (left) and electrochemically reduced (right) states.

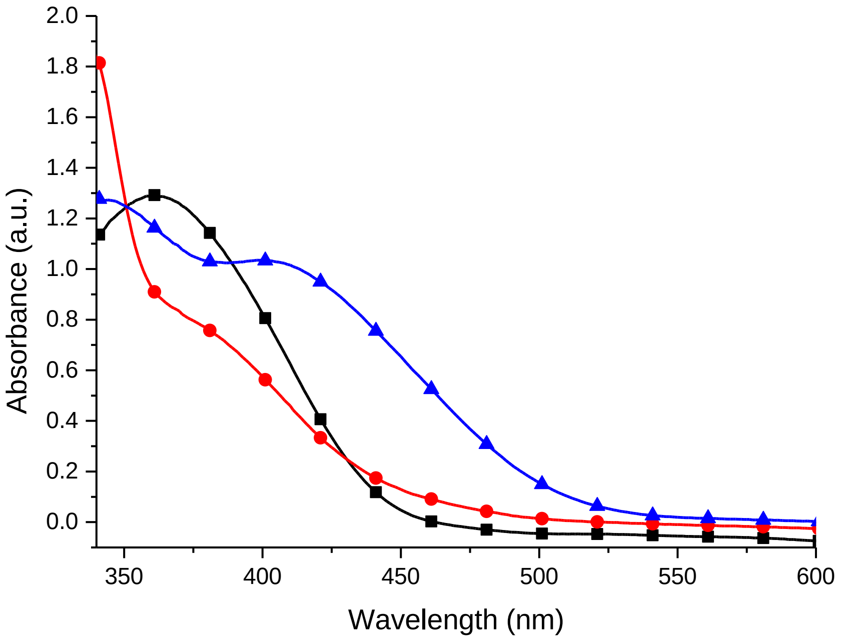

Figure S5. The absorption spectra of **poly-1** in its neutral Fe(II) state (■), oxidized Fe(III) state at +1.0V (●) and reduced Fe(I) state (▲) at −0.5V.

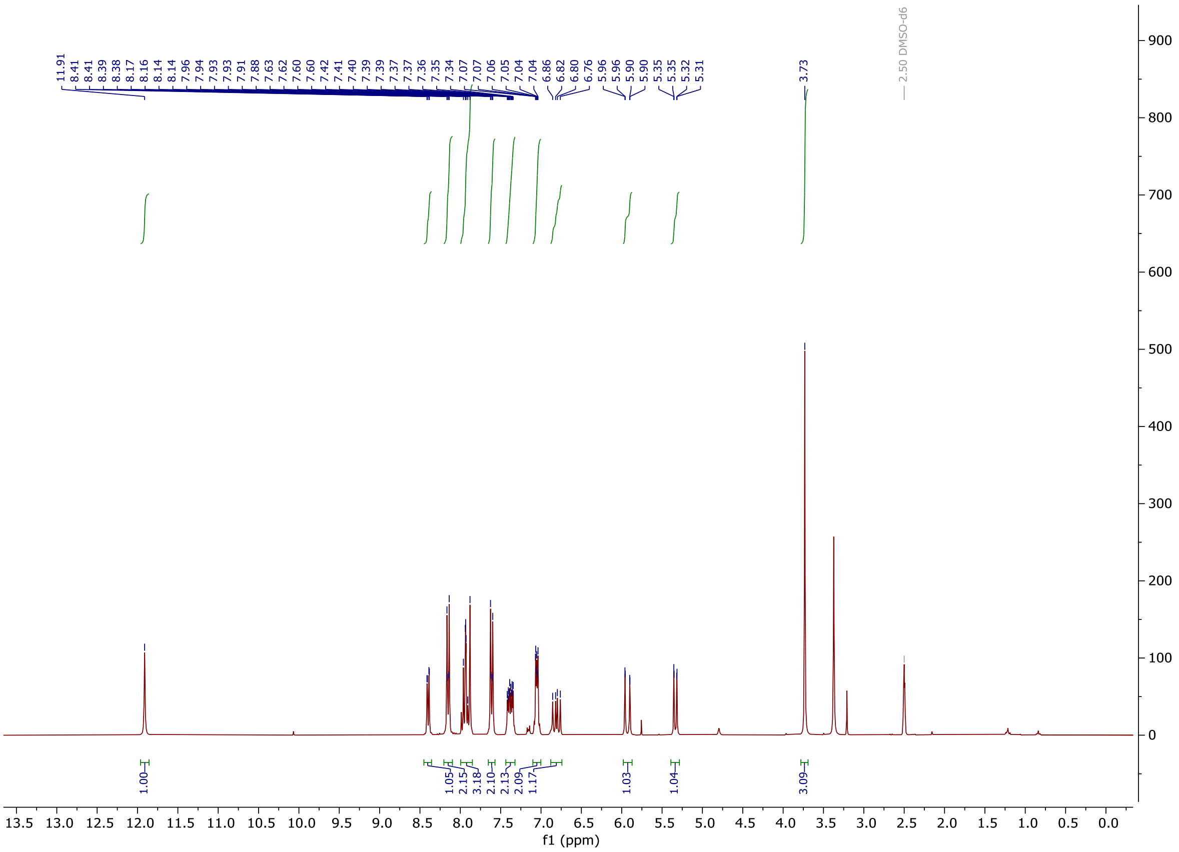

Figure S6. ^{1}H NMR of ligand **L** in d_6-DMSO.

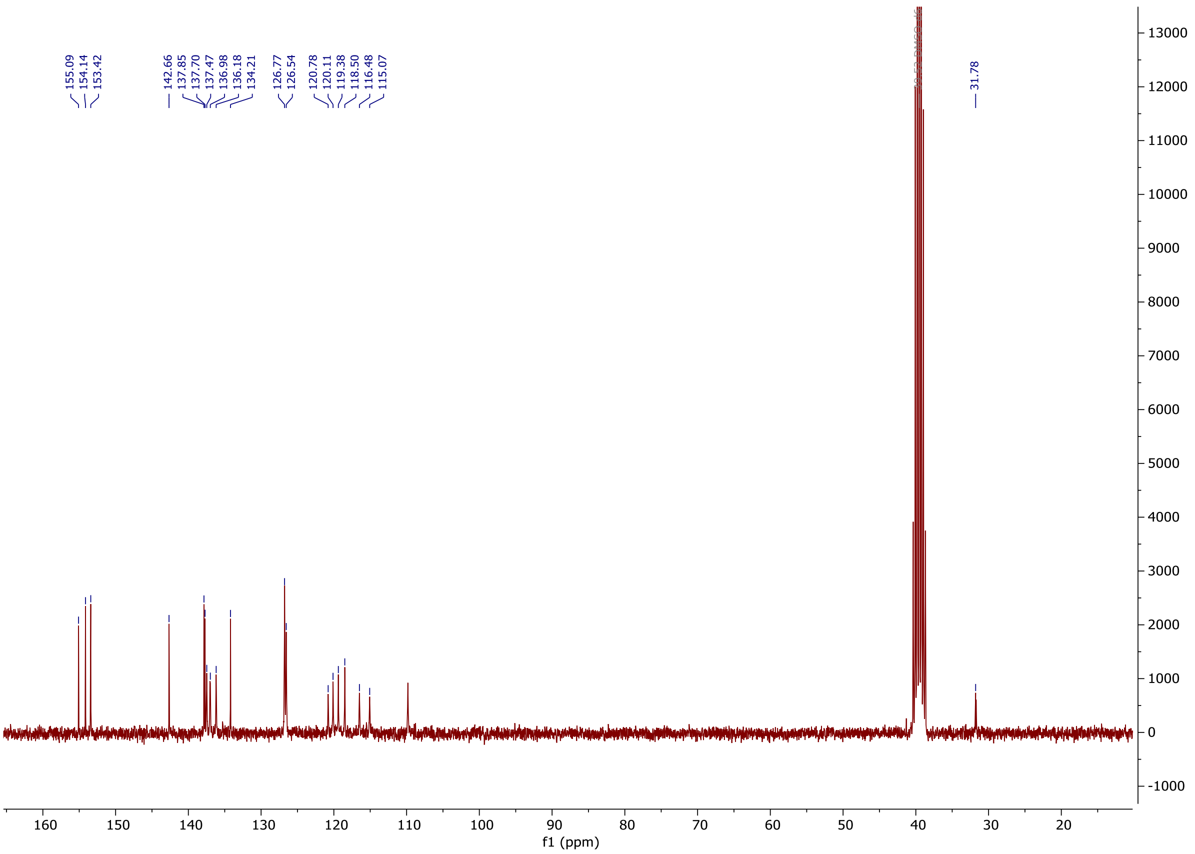

Figure S7. ^{13}C NMR spectra of ligand **L** in d_6-DMSO.

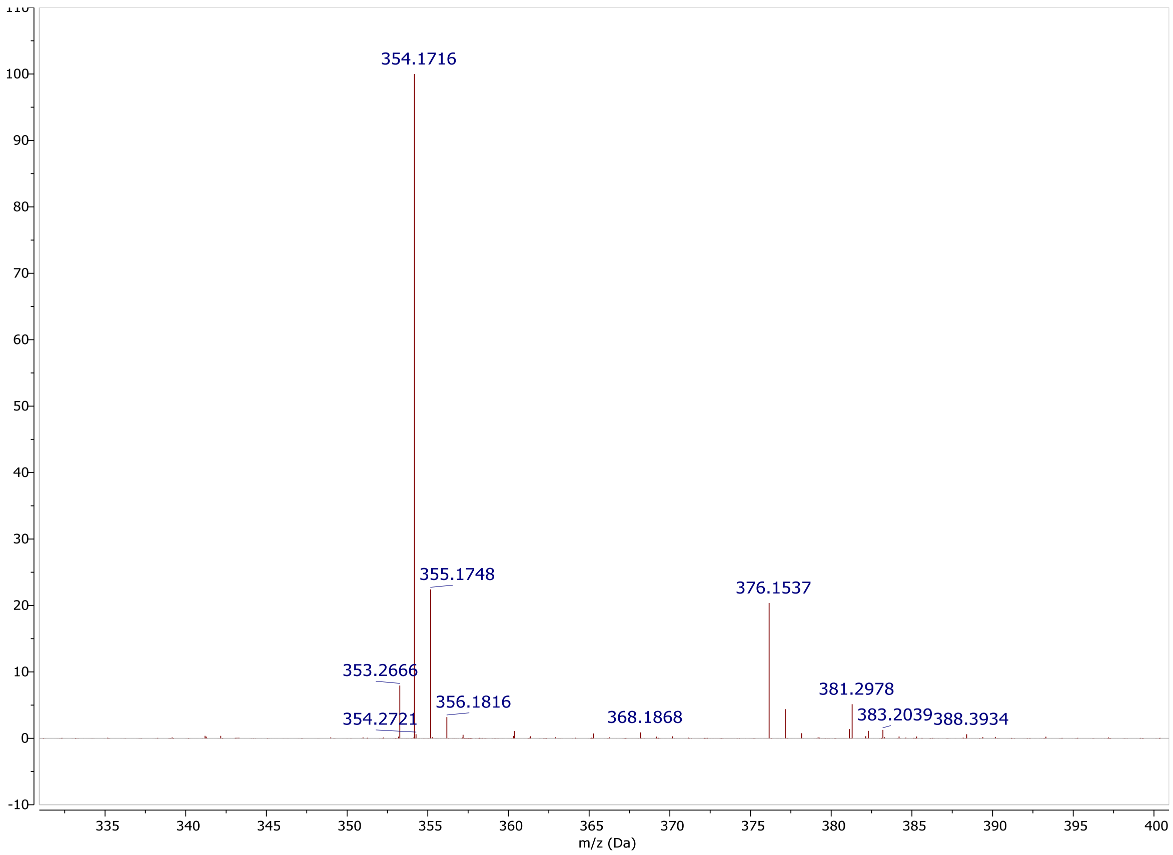

Figure S8. HR-MS spectra of ligand **L**.

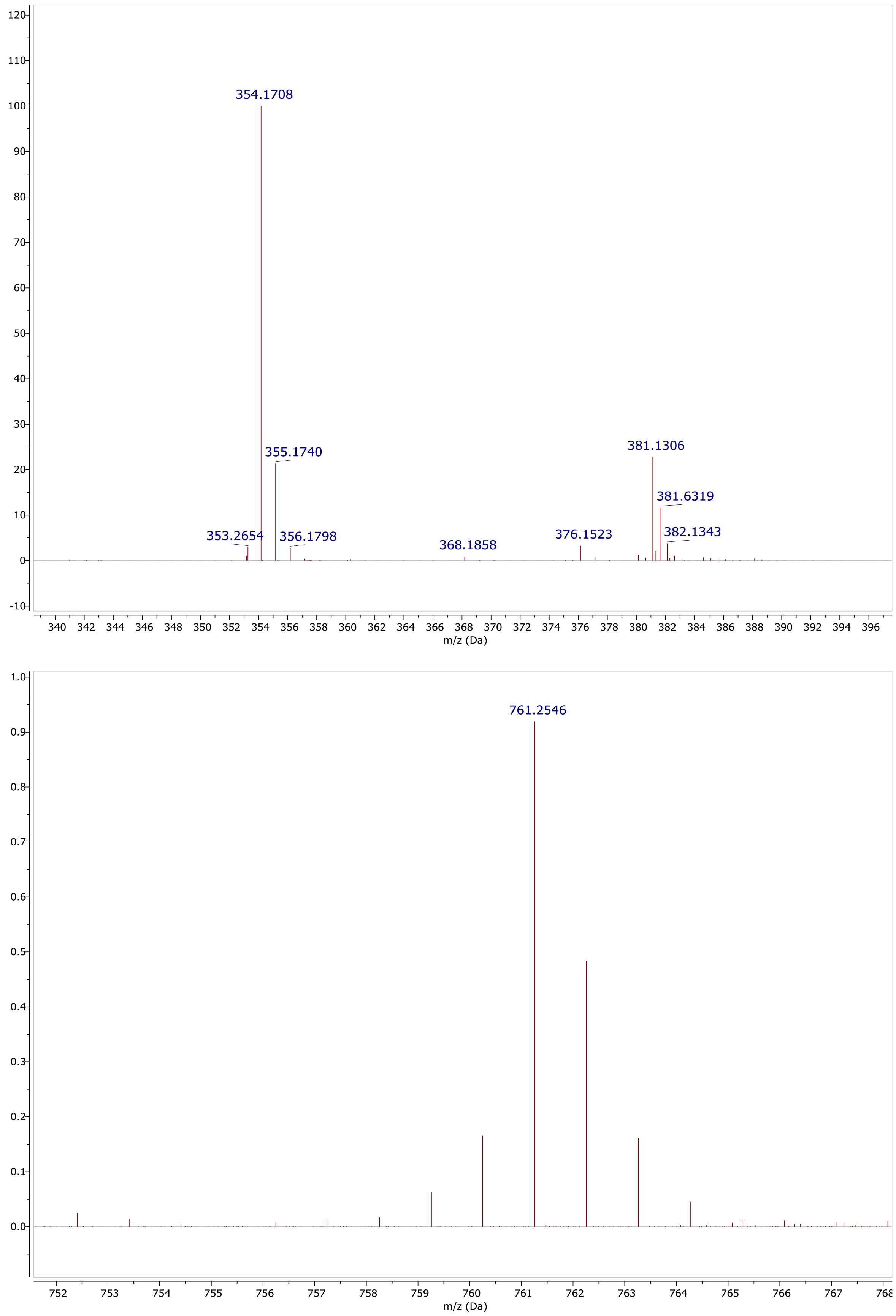

Figure S9. ESI-MS spectra of complex **1**.

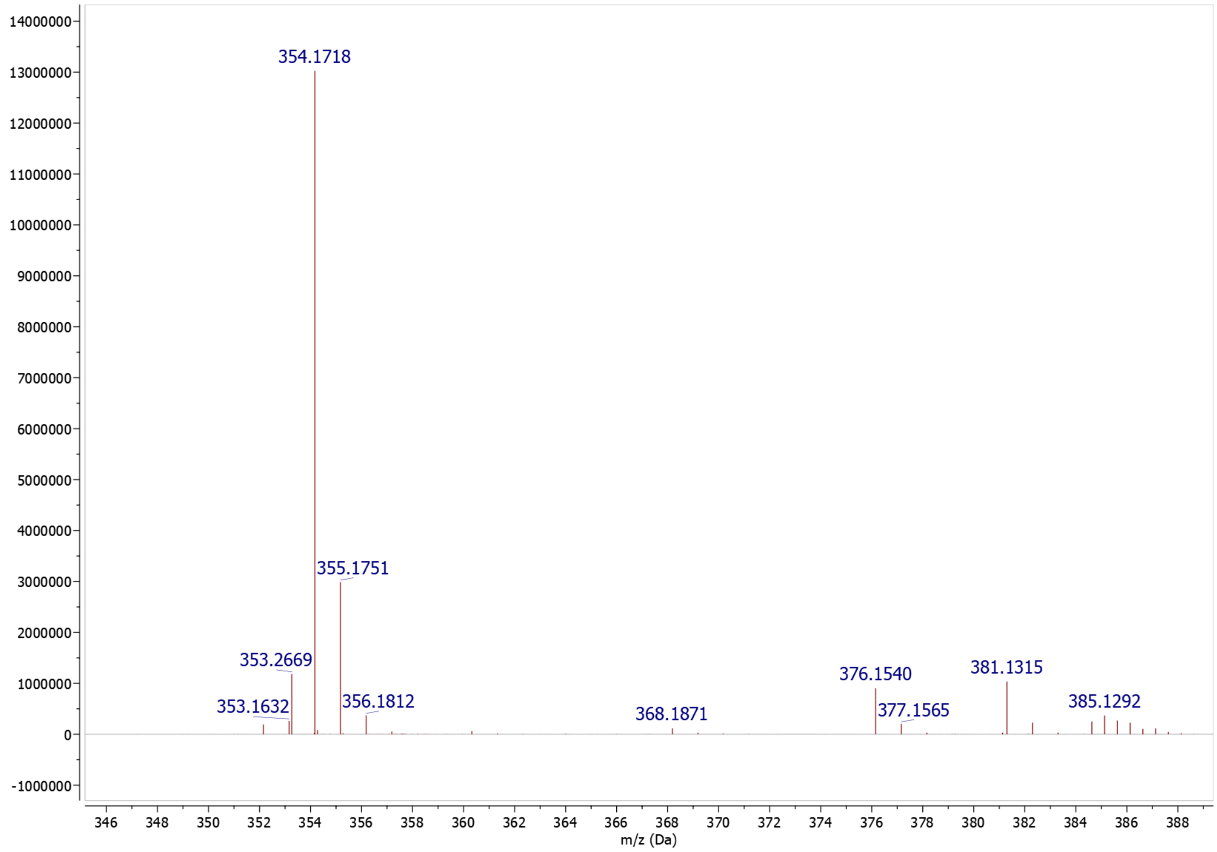

Figure S10. ESI-MS spectra of complex **2**.
